# Supplementary material for: Bose-Einstein condensation of non-ground-state caesium atoms
Source: Nat Commun. 2024 May 3;15:3739. doi: 10.1038/s41467-024-47760-0 (PMC11068738; doi:10.1038/s41467-024-47760-0)
Supplement: Supplementary file 1 — Supplementary Information [file 41467_2024_47760_MOESM1_ESM.pdf]

Bose-Einstein condensation of non-ground-state caesium atoms
Supplementary materials

Milena Horvath,[1] Sudipta Dhar,[1] Arpita Das,[2] Matthew D. Frye,[3] Yanliang Guo,[1] Jeremy M. Hutson,[3] Manuele Landini,[1] and Hanns-Christoph Nägerl[1,*]

[1] *Institut für Experimentalphysik und Zentrum für Quantenphysik, Universität Innsbruck, Technikerstraße 25, Innsbruck, 6020, Austria*
[2] *Joint Quantum Centre (JQC) Durham-Newcastle, Department of Physics, Durham University, Durham DH1 3LE, United Kingdom*
[3] *Department of Chemistry and Joint Quantum Centre (JQC) Durham-Newcastle, Durham University, Durham DH1 3LE, United Kingdom*

I. SUPPLEMENTARY NOTE 1: CALCULATED RESONANCE PARAMETERS

We locate and characterise resonances in our coupled-channel calculations using the methods of Ref. [1]. We use the regularised scattering length procedure, which is suitable for the weak background inelasticity that is present for the resonances here. Characterising the d-wave resonance in Fig. 3 near 147.5 G is complicated by the significant variation of the background scattering length $a_{\rm bg}$ across its width. We therefore subtract off a field-dependent reference $a_{\rm ref}(B) = (B - 147.5\ {\rm G}) \times 25\ a_0\ {\rm G}^{-1}$ before fitting to obtain the parameters given in the main text. The field-variation of the background means it is not possible to define the resonance width Δ as usual, but the strength $a_{\rm bg}\Delta$ is nonetheless well defined. The g-wave resonance shown in Fig. 3 is narrower and simpler to fit, giving parameters $B_{\rm res} = 154.18$ G, $\Delta = 20$ mG, $a_{\rm bg} = 160\ a_0$, and $a_{\rm res} = 3.2 \times 10^6\ a_0$.

The resonances described in Supplementary Note II are harder to assign and characterise. The coupled-channel calculations described in the main text use the interaction potential of Berninger *et al.* [2] with a basis set limited by $L_{\rm max} = 4$. This is appropriate because the potential was fitted to experimental results using this basis set, so that the potential itself accounts (in an averaged way) for the absence of basis functions with $L > 4$. However, these calculations show only the two resonances discussed above in the region of interest. We therefore carry out further calculations with $L_{\rm max} = 6$ and 8. These calculations reveal several additional narrow resonances in this region, due to i-wave ($L = 6$) states; their parameters are listed in Supplementary Table I. In each case there is at least one i-wave resonance within 3 G of the observed loss feature, but there is no clear mapping between the individual calculated resonances and experimental loss peaks. Specific assignments of the loss peaks would require refitting the entire interaction potential, using a more accurate form for the shorter-range part of the interaction potential than in Ref. [2]. This is outside of the scope of the present work.

II. SUPPLEMENTARY NOTE 2: RESONANCES IN THE RANGE OF B = 138 TO 145 G

We carry out an atomic loss spectroscopy measurement in the magnetic field region between 138 and 145 G by holding a non-condensed sample of atoms in $(3, 2)$ in the dipole trap for 2 s and recording the number of remaining

$B_{\rm res}$ (G)	Δ (mG)	$a_{\rm bg}$ (a_0)	$a_{\rm res}$ (a_0)
134.28	−0.0076	−402	2.5×10^4
136.96	−0.12	−293	3.3×10^4
141.10	−0.47	−147	1.7×10^6
144.99	−4.1	−21.3	107
147.73	−2.9	−154	5.9×10^6

SUPPLEMENTARY TABLE I. Parameters of i-wave Feshbach resonances between 130 and 150 G, from coupled-channel calculations with $L_{\rm max} = 8$.

* christoph.naegerl@uibk.ac.at

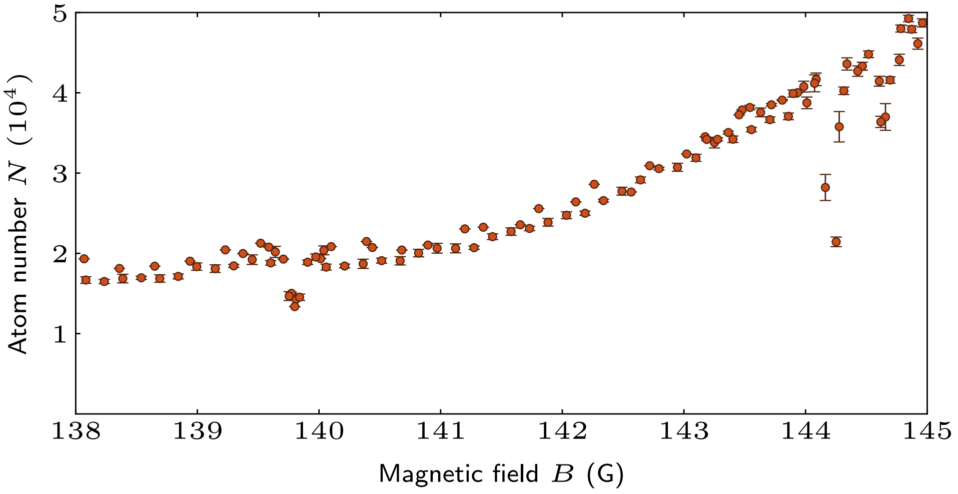

SUPPLEMENTARY FIG. 1. **Search for additional Feshbach resonances.** Loss spectroscopy performed with a non-condensed cloud in the dipole trap between 138 G and 145 G. For this measurement the sample is held in the trap for 2 s before imaging via the standard TOF technique. Each data point is an average of up to five repetitions.

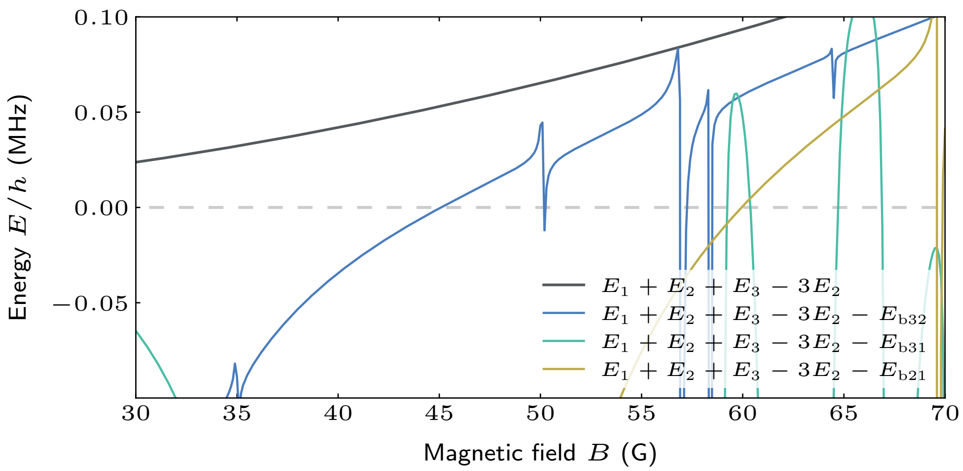

SUPPLEMENTARY FIG. 2. **Energies of possible decay channels for spin-exchange-assisted three-body recombination around 40 G.** Excess Zeeman energy required for the spin-exchange process $(3,2)+(3,2) \to (3,3)+(3,1)$ (black). Energy difference between the excess Zeeman energy and the binding energy of a weakly bound dimer in $(3,3)+(3,2)$ (blue), $(3,3)+(3,1)$ (green) and $(3,2)+(3,1)$ (yellow). In the legend the Zeeman shift and the binding energies are labeled as E_{m_f} and $E_{\mathrm{b},m_{f1},m_{f2}}$, respectively. The dashed line shows zero energy separation.

atoms N as B is varied. The results are plotted in Supplementary Fig. 1. We find three loss features within this region, at 139.80(1), 144.48(1) and 144.65(1) G.

III. SUPPLEMENTARY NOTE 3: THREE-BODY RECOMBINATION PROCESSES ASSISTED BY SPIN EXCHANGE

As a possible collision process accounting for the large three-body loss measured in the magnetic field region around 40 G, we consider three-body recombination assisted by a spin-exchange process. We begin with three atoms in the $(3,2)$ state, each with energy E_2, undergoing a spin-exchange collision that produces one atom in each of the states

$(3,3)$, $(3,2)$ and $(3,1)$, with corresponding energy E_{m_f}. Between 40 and 50 G, the Zeeman energy, mostly dominated by the quadratic Zeeman shift, results in an excess energy of approximately 50 to 100 kHz for this spin-exchange process. This is of the same order of magnitude as the binding energy $E_{\mathrm{b},m_{f1},m_{f2}}$ of a weakly bound dimer in the channel $(3,3)+(3,2)$ in this region of field. In Supplementary Fig. 2 we show the energy difference between the excess Zeeman energy and the binding energy of weakly bound dimers in the spin channels $(3,3)+(3,2)$, $(3,3)+(3,1)$, and $(3,2)+(3,1)$, respectively. The binding energies of the dimers are calculated using

$$E_{\mathrm{b},m_{f1},m_{f2}} = -\hbar^2/(2\mu(a_{m_{f1},m_{f2}} - \bar{a})^2), \quad (1)$$

where μ is the reduced mass of the atom pair and $\bar{a} \approx 96.56a_0$ is the mean scattering length of Cs. From Supplementary Fig. 2 we see that at 45 G the formation of a molecule in the $(3,3)+(3,2)$ channel becomes energetically resonant. This suggests that three-body recombination via a spin-exchange collision is the likely cause of the large values of k_3 that we measure in this region. Interestingly we further find from Supplementary Fig. 2 that molecular channels $(3,2)+(3,1)$ and $(3,3)+(3,1)$ also have resonant features above 55 G.

This process differs from a typical three-body recombination collision, where the molecular binding energy is carried away by the collision products. Here most of the binding energy is absorbed by the excess Zeeman energy, so the products do not gain significant kinetic energy.

[1] M. D. Frye and J. M. Hutson, Characterizing Feshbach resonances in ultracold scattering calculations, Phys. Rev. A **96**, 042705 (2017).

[2] M. Berninger, A. Zenesini, B. Huang, W. Harm, H.-C. Nägerl, F. Ferlaino, R. Grimm, P. S. Julienne, and J. M. Hutson, Feshbach resonances, weakly bound molecular states, and coupled-channel potentials for cesium at high magnetic fields, Phys. Rev. A **87**, 032517 (2013).
